# Effective PID controller design using a novel hybrid algorithm for high order systems

**DOI:** 10.1371/journal.pone.0286060

**Published:** 2023-05-26

**Authors:** Davut Izci, Serdar Ekinci, Abdelazim G. Hussien

**Affiliations:** 1 Department of Computer Engineering, Batman University, Batman, Turkey; 2 MEU Research Unit, Middle East University, Amman, Jordan; 3 Department of Computer and Information Science, Linköping University, Linköping, Sweden; 4 Faculty of Science, Fayoum University, Fayoum, Egypt; J.C. Bose University of Science and Technology, YMCA, INDIA, INDIA

## Abstract

This paper discusses the merging of two optimization algorithms, atom search optimization and particle swarm optimization, to create a hybrid algorithm called hybrid atom search particle swarm optimization (h-ASPSO). Atom search optimization is an algorithm inspired by the movement of atoms in nature, which employs interaction forces and neighbor interaction to guide each atom in the population. On the other hand, particle swarm optimization is a swarm intelligence algorithm that uses a population of particles to search for the optimal solution through a social learning process. The proposed algorithm aims to reach exploration-exploitation balance to improve search efficiency. The efficacy of h-ASPSO has been demonstrated in improving the time-domain performance of two high-order real-world engineering problems: the design of a proportional-integral-derivative controller for an automatic voltage regulator and a doubly fed induction generator-based wind turbine systems. The results show that h-ASPSO outperformed the original atom search optimization in terms of convergence speed and quality of solution and can provide more promising results for different high-order engineering systems without significantly increasing the computational cost. The promise of the proposed method is further demonstrated using other available competitive methods that are utilized for the automatic voltage regulator and a doubly fed induction generator-based wind turbine systems.

## Introduction

Metaheuristic algorithms comprise a diverse range of approaches, each with their unique strengths and limitations [1]. These algorithms are inspired by physical and biological phenomena and are well-suited to solving complex optimization problems that traditional methods struggle to handle [2]. With their straightforward and adaptable structure, coupled with the ability to avoid getting stuck in local optima through random search, metaheuristics have become increasingly popular for tackling a wide range of problems [3–10]. In recent years, these techniques have become a prominent area of research for solving high-order real-world problems, thanks to their ability to provide efficient and optimal solutions with ease of implementation, making them a go-to tool for a wide range of engineering design applications [11–19]. Since metaheuristics treat the problem as a black box and attempt to solve it without any knowledge of its nature, they are highly applicable to engineering optimization problems.

It is crucial to note, however, that no single algorithm can optimally solve all problems, as each approach has its own set of advantages and disadvantages. The "no free lunch theorem" [20] states that there is no one-size-fits-all algorithm. As per this theorem, the atom search optimization algorithm [21] was created to tackle optimization problems and has so far been implemented in a variety of engineering problems from designing power system stabilizer [22] to regulating the speed of a direct current motor and training multi-layer perceptron [23]. More examples can also be encountered from related literature [24–28]. Inspired by the movement of atoms in nature, atom search optimization employs interaction forces such as attraction and repulsion, as well as a constraint force, to guide each atom in the population. The better-performing atoms experience a smaller acceleration, while the weaker ones experience a larger acceleration [21]. Atom search optimization’s neighbor interaction also plays a vital role in the optimization process. During the early iterations, a larger number of atoms are used to explore the search space entirely, while in later iterations, less atoms are utilized for the optimal solution exploitation [29]. This approach allows for a seamless exploration-exploitation transition and results in a highly effective exploration ability.

Despite its many advantages, atom search optimization does have some limitations, including weak exploitation, the risk of getting stuck in local optima, and a lack of proper exploration-exploitation balance [30]. One way to enhance the performance of atom search optimization is to combine it with other optimizers to improve the exploration and exploitation abilities. For instance, Barshandeh and Haghzadeh [30] combined tree-seed algorithm and Levy flight to promote exploration and exploitation. Abd Elaziz et al. [28] integrated sine-cosine algorithm as a local search operator to enhance exploration. Jadhav and Joshi [31] incorporated sunflower optimization to improve search ability, while Eker et al. [23] added simulated annealing to improve local search capability.

However, it is important to note that hybridizing two algorithms may lead to increased computational cost, particularly for high-dimensional real-world engineering problems [32]. Additionally, during the optimization process, the constants in the hybrid version need to adaptively be tuned to avoid the loss of exploration. Hybrid algorithms should also focus on strengthening the exploration of the algorithms in the later iterations. Therefore, combining two or more algorithms into one component is a useful strategy to improve algorithm performance while reducing computational cost. In this context, the performance of the atom search optimization can also be improved by incorporating stochastic operators. For example, Menaga and Revathi [33] integrated fractional calculus into atom search optimization to develop fractional-based version of it, which effectively improved the search effectiveness. Rizk-Allah et al. [34] proposed an improved version of atom search optimization by using three strategies, including random search, information-based extended, and trimming strategies, to enhance the quality of solutions. Further examples such as chaotic maps [35] and Euclidian distance ratio [24] can also be encountered in the literature. These stochastic operators provide an alternative to hybridizing atom search optimization with other optimizers and can enhance the optimization performance without significantly increasing the computational cost.

Improved versions of atom search optimization have been proposed in the literature, indicating the feasibility of designing more efficient algorithms. However, achieving a balance between exploration and exploitation remains a challenge. Thus, there is a need to establish an effective balance strategy for optimization problems [3]. One major research attempt to achieve this goal is to combine the advantages of different algorithms into a single component, while introducing adaptive strategies to balance exploration and exploitation [36], which is the idea behind the work carried out in this study. In line with this motivation, a new hybrid approach that combines the strengths of atom search optimization and particle swarm optimization has been developed in this study. Particle swarm optimization has a fast convergence speed, but it tends to converge prematurely on local optima when solving optimization problems [37]. Due to its simple structure, ease of implementation, and advantages, the particle swarm optimization is often used as an effective component to enhance the performance of other algorithms [38–40].

The proposed algorithm, called hybrid atom search particle swarm optimization, aims to achieve a more effective balance between exploration and exploitation stages, leading to improved search efficiency. To achieve this, the global search capability of atom search optimization is combined with the ability of social thinking in particle swarm optimization. The efficacy of the constructed algorithm has been used to improve the time-domain performance of two different high-order real-world engineering problems. In this regard, the design of a proportional-integral-derivative controller for an automatic voltage regulator [41] and a doubly fed induction generator-based wind turbine system [42] are considered in this paper in order to demonstrate the superior performance of the proposed method. Comparing the results with other approaches in the literature, it is evident that the hybrid atom search particle swarm optimization outperforms them in tuning controller parameters for different high-order systems, demonstrating its superiority and potential for future optimization applications. The major contributions of our work can be listed as:

- Combining the global search capability of atom search optimization with the ability of social thinking in particle swarm optimization for improved search efficiency.
- Demonstrating the efficacy of the proposed algorithm in improving the time-domain performance of two different high-order real-world engineering problems, namely the design of a proportional-integral-derivative controller for an automatic voltage regulator and a doubly fed induction generator-based wind turbine system.
- Emphasizing the need to establish an effective balance strategy for optimization problems and presenting a promising approach to achieving this goal through the combination of different algorithms into a single component with adaptive strategies to balance exploration and exploitation.

The rest of the paper is organized in such a way to ensure that it is presented in a logical and coherent manner, allowing readers to follow the arguments and conclusions easily. In this regard, the following section describes the basic principles of atom search optimization, discusses its advantages and limitations, and introduces the hybrid atom search particle swarm optimization algorithm. The third section presents the mathematical models of the automatic voltage regulator and doubly fed induction generator-based wind turbine systems. This is followed by another section providing an overview of proportional-integral-derivative controllers and their applications in control systems. The fifth section outlines the steps involved in the proposed design procedure and explains how the proposed algorithm is used to optimize the design parameters of the adopted controller. The sixth section presents the comparative results of the automatic voltage regulator and doubly fed induction generator-based wind turbine systems, analyzes the simulation results, and provides insights into the performance of the proposed design procedure. The paper is finally concluded in the seventh section by summarizing the main findings of the study, discussing the implications and significance of the proposed design procedure, identifying the limitations of the study, and suggesting avenues for future research.

### Atom search optimization and proposed algorithm

#### Standard atom search optimization

The atom search optimizer (ASO) is a global optimization technique that draws inspiration from molecular dynamics, as outlined in Zhao et al. [43]. In essence, the algorithm represents the movement of atoms according to classical mechanics through mathematical equations. Specifically, the relationship between atoms in a system stems from Newton’s second law, where the force of interaction (*F*_*i*_) and constraint (*G*_*i*_) acting on a given atom (*i*) are taken into account. The following is a mathematical demonstration of this relationship [43].


$$a_{i}=\frac{F_{i}+G_{i}}{m_{i}}$$ (1)

Eq (1) uses *a*_*i*_ to represent the acceleration of atom *i*, while *m*_*i*_ refers to the mass of the same atom. The interaction force between two atoms, *i* and *j*, can be expressed using the following equation in *d* dimensions and at time *t* [44].


$$F_{ij}^{\prime }(t)=-\eta (t)\left[2{\left(h_{ij}(t)\right)}^{13}-{\left(h_{ij}(t)\right)}^{7}\right]$$ (2)

In here, *η*(*t*) represents the depth function (the depth weight for this function was taken as 50 for this study) which is used to adjust repulsion or attraction regions [22] whereas *h*_*ij*_(*t*) is described with the following form [43]:

$$h_{ij}(t)=\left\{ \begin{array}{c} h_{min},\;\frac{r_{ij}(t)}{\sigma (t)}<h_{min}\\ \frac{r_{ij}(t)}{\sigma (t)},\;h_{min}\leq \frac{r_{ij}(t)}{\sigma (t)}\leq h_{max}\\ h_{max},\;\frac{r_{ij}(t)}{\sigma (t)}>h_{max} \end{array}\right.$$ (3)

The function *h*_*ij*_(*t*) represents the distance between two atoms (*r*), lower bound (*h*_*min*_), and upper bound (*h*_*max*_). This function helps to determine equilibrium, repulsion, or attraction between atoms. The terms *g*_0_ and *u* are set to 1.1 and 1.24, respectively, to represent the limits and the collision diameter is denoted by *σ*(*t*) [22]. The following expression is employed by this algorithm to drift from exploration to exploitation [43].


$$g(t)=0.1\times \sin\left(\frac{\pi }{2}\times \frac{t}{T}\right)$$ (4)

In *d*^*th*^ dimension, the total force exerting on atom *i* is given as follows where *rand*_*j*_ represents a random number within [0, 1].


$$F_{i}^{d}(t)=\sum_{j\in Kbest}{rand}_{j}F_{ij}^{d}(t)$$ (5)

The constraint force is described as $G_{i}^{d}(t)=\lambda (t)\left(x_{best}^{d}(t)-x_{i}^{d}(t)\right)$ where the best atom position in iteration *t* is denoted by *x*_*best*_(*t*) and $\lambda (t)=\beta e^{-\frac{20t}{T}}$. In here, *β* denotes the multiplier weight and it is taken as 0.2 for this study. The *i*^*th*^ atom’s acceleration at time *t* is described as follows where the mass of *i*^*th*^ atom at time *t* is represented by *m*_*i*_(*t*) [43].


$$a_{i}^{d}(t)=\frac{F_{i}^{d}(t)}{m_{i}^{d}(t)}+\frac{G_{i}^{d}(t)}{m_{i}^{d}(t)}$$ (6)

To compute the mass of the *i*^*th*^ atom, $m_{i}(t)=M_{i}(t)/\sum_{j=1}^{N}M_{j}(t)$ is used where *M*_*i*_(*t*) is given as follows [43].


$$M_{i}(t)=e^{-\frac{{Fit}_{i}(t)-{Fit}_{best}(t)}{{Fit}_{worst}(t)-{Fit}_{best}(t)}}$$ (7)

In here, *Fit*_*best*_(*t*), *Fit*_*worst*_(*t*) and *Fit*_*i*_(*t*) denote the atoms with the minimum fitness value, the maximum fitness value and function fitness value of atom *i* at iteration *t*. To improve exploration, it’s necessary to interact with atoms that have higher fitness values as neighbors. To simplify the structure of the ASO algorithm, the followings are used for the velocity and position of the *i*^*th*^ atom position at iteration (*t*+1) [44].


$$\nu_{i}^{d}(t+1)={rand}_{i}^{d}\nu_{i}^{d}(t)+a_{i}^{d}(t)$$ (8)

$$x_{i}^{d}(t+1)=x_{i}^{d}(t)+\nu_{i}^{d}(t+1)$$ (9)

On the other hand, to enhance exploitation, the number of these interactions should be reduced. The value of *K* (the interaction possibility of the atoms with better fitness values as each atom’s *K* neighbors) is calculated as $K(t)=N-(N-2)\times \sqrt{t/T}$ [44]. The next subsection describes the structure of the proposed hybrid atom search particle swarm optimization technique.

### Hybrid atom search particle swarm optimization

It is feasible to encounter several hybridization techniques in literature related to metaheuristic algorithms [45]. It is feasible to hybridize two algorithms in two ways: high-level and low-level, using either relay or co-evolutionary methods, and they can be either homogeneous or heterogeneous [46]. In this study, a low-level coevolutionary heterogeneous hybrid approach is adopted to combine ASO and particle swarm optimization (PSO) algorithms. The hybrid method is considered low-level because it integrates the functionality of both algorithms. It is also co-evolutionary since both algorithms are run in parallel, rather than sequentially. Moreover, it is heterogeneous because two different algorithms are utilized to generate the final results. The idea behind the proposed hybrid atom search particle swarm optimization (h-ASPSO) algorithm is to combine the global search capability of ASO with the ability of social thinking (*g*_*best*_) in PSO. In this context, the following equations are proposed to update the velocity and the position.

$$v_{i}(t+1)=w\times v_{i}(t)+c_{1}\times rand\times a_{i}(t)+c_{2}\times rand\times (g_{best}-x_{i}(t))$$ (10)

$$x_{i}(t+1)=x_{i}(t)+v_{i}(t+1)$$ (11)

where *g*_*best*_ is the best solution so far, *a*_*i*_(*t*) is the acceleration of the *i*^*th*^ agent at iteration *t*, *w* is a weighting function having the expression of $w=w_{max}-(w_{max}-w_{min})\times (t/t_{max})$ where *t*_*max*_ is the total iteration number. The values of the *w*_*max*_ and *w*_*min*_ are set as *w*_*max*_ =0.9 and *w*_*min*_ = 0.4 for optimum performance. *c*_1_ and *c*_2_ are the weighting factors which are set to *c*_1_=0.5 and *c*_2_= 1.5.

**Fig 1:**
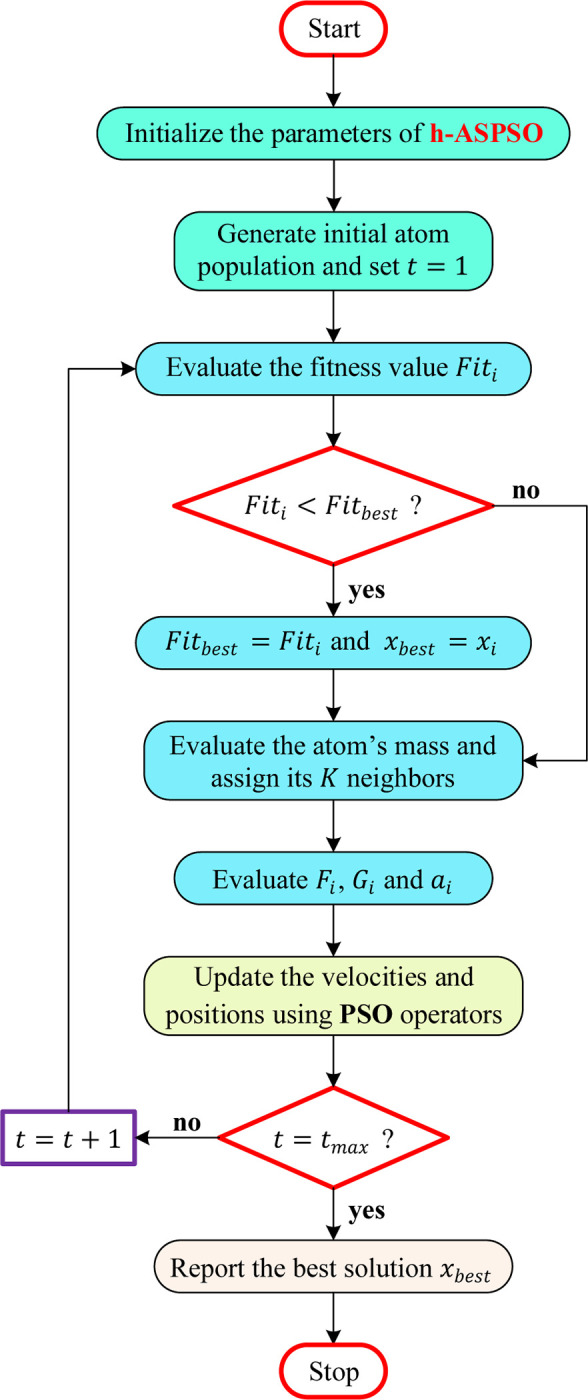
Flowchart for h-ASPSO.

In h-ASPSO, the parameters are initialized first and the initial atom population is set. The agents in the population are considered as candidate solutions. After initialization, standard ASO algorithm performs for evaluation of *F*_*i*_, *G*_*i*_ and *a*_*i*_, respectively. After this stage the PSO related operators are used to update the velocities and positions as described in Eqs (10) and (11), respectively. This procedure is followed until the end of iterations (stopping criteria). The flowchart in Fig 1 displays the steps of the hybrid h-ASPSO algorithm proposed in this study. This algorithm is used to effectively tune the parameters of a proportional-integral-derivative controller adopted in two high-order engineering systems known as automatic voltage regulator and doubly fed induction generator-based wind turbine. The following section describes the nature of those systems.

### Modeling the high-order engineering systems

The following subsections under this part of the paper respectively provide mathematical models of the automatic voltage regulator and double-fed induction generator-based wind turbine systems in order to set the basis for the optimization procedures.

### Mathematical model of automatic voltage regulator

**Fig 2:**
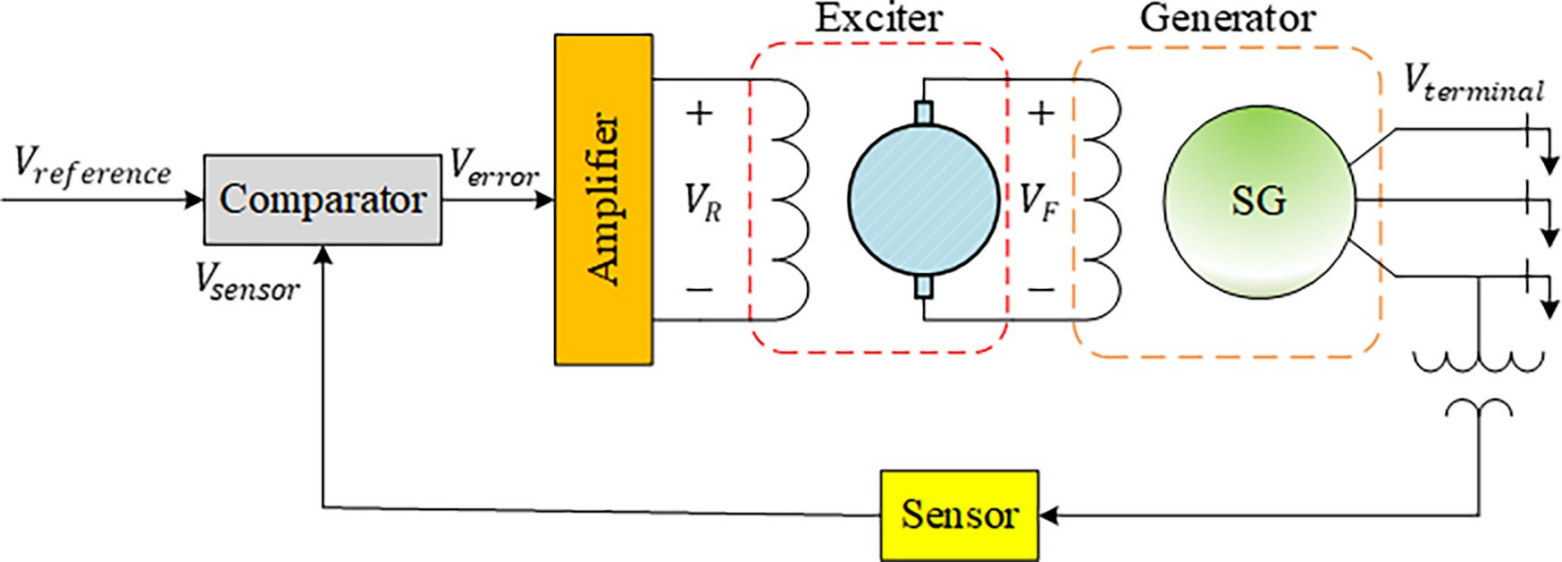
Schematic diagram of a basic AVR system.

The output voltage of a generator in a power system is controlled and regulated by a device named automatic voltage regulator (AVR). The AVR typically uses electronic components such as amplifiers, exciters, generators, and sensors to maintain a constant output voltage [47–49]. Those components are presented in the schematic diagram given in Fig 2.

The AVR plays a critical role in maintaining voltage stability as electrical equipment can be damaged or fail to operate without proper voltage regulation which would lead to serious failures. The AVR is therefore an essential component of power systems that rely on generators or alternators for electricity generation. The transfer function of an AVR system can be described as follows [50].


$$G(s)=\frac{0.1s+10}{0.0004s^{4}+0.0454s^{3}+0.555s^{2}+1.51s+11}$$ (12)

**Fig 3:**
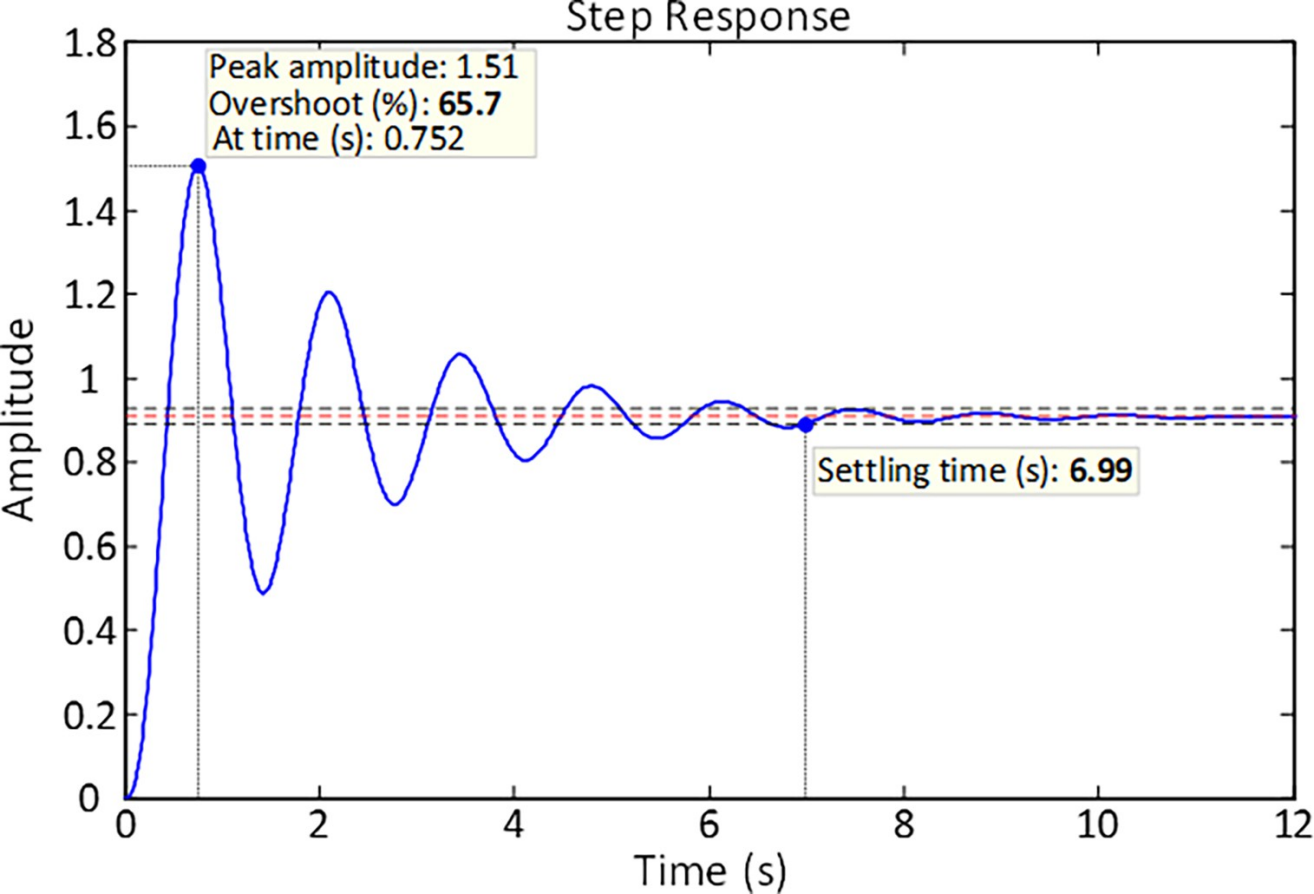
Step response of uncontrolled AVR system.

The step response in Fig 3 belongs to an uncontrolled AVR system illustrating that although the system is stable, it exhibits significant oscillation. The generator terminal voltage step response reaches an overshoot of 65.7% and the system has a settling time of 6.99 *s*. Lack of control in an AVR system leads to significant oscillation in the transient state response and an error in the steady state which are not acceptable reactions in power systems.

### Mathematical model of wind turbine system

**Fig 4:**
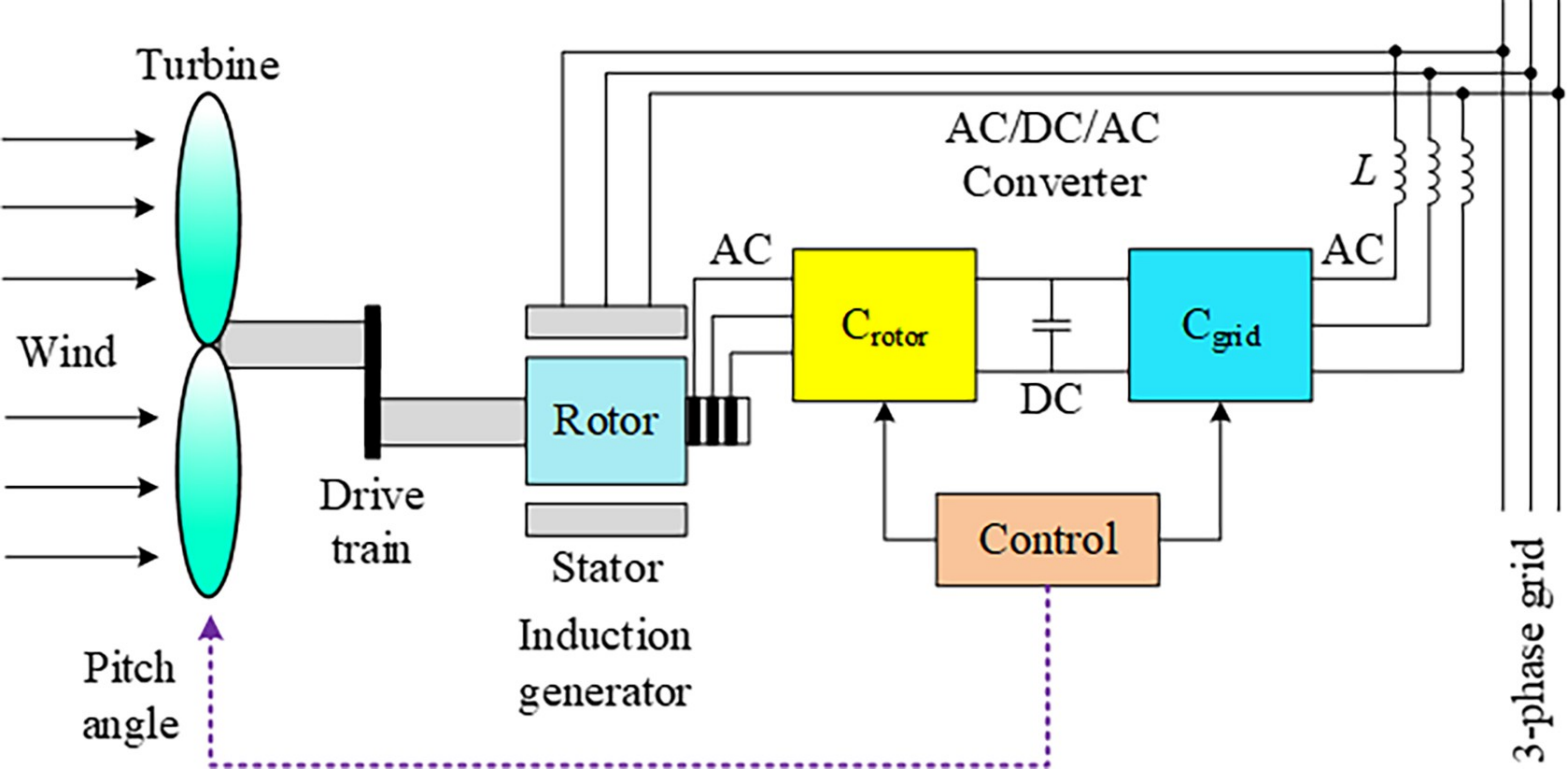
Schematic diagram of a DFIG-based wind turbine system.

In Fig 4, a simplified illustration of the wind turbine system based on the doubly fed induction generator (DFIG) is presented. This state-of-the-art system includes several components, such as the wind turbine, drive train, induction generator, AC/DC/AC converters, and power transformer. The DFIG-based wind turbine system is designed to harness the wind’s potential energy in two stages. First, it cleverly converts the wind power into mechanical power, and then this mechanical energy is further converted into sustainable, clean, and efficient electrical power. Overall, it is a remarkable system that showcases the enormous potential of renewable energy sources [51].

The following 6^*th*^ order transfer function represents the transient behavior of the system.


$$G(s)=\frac{0.000324s^{6}-1.75s^{5}-2366s^{4}+7.9e06s^{3}+7.5e09s^{2}+5e12s+2.18e14}{s^{6}+2340s^{5}+8.67e06s^{4}+4.79e09s^{3}+2.7e12s^{2}+1.27e14s+9.6e14}$$ (13)

**Fig 5:**
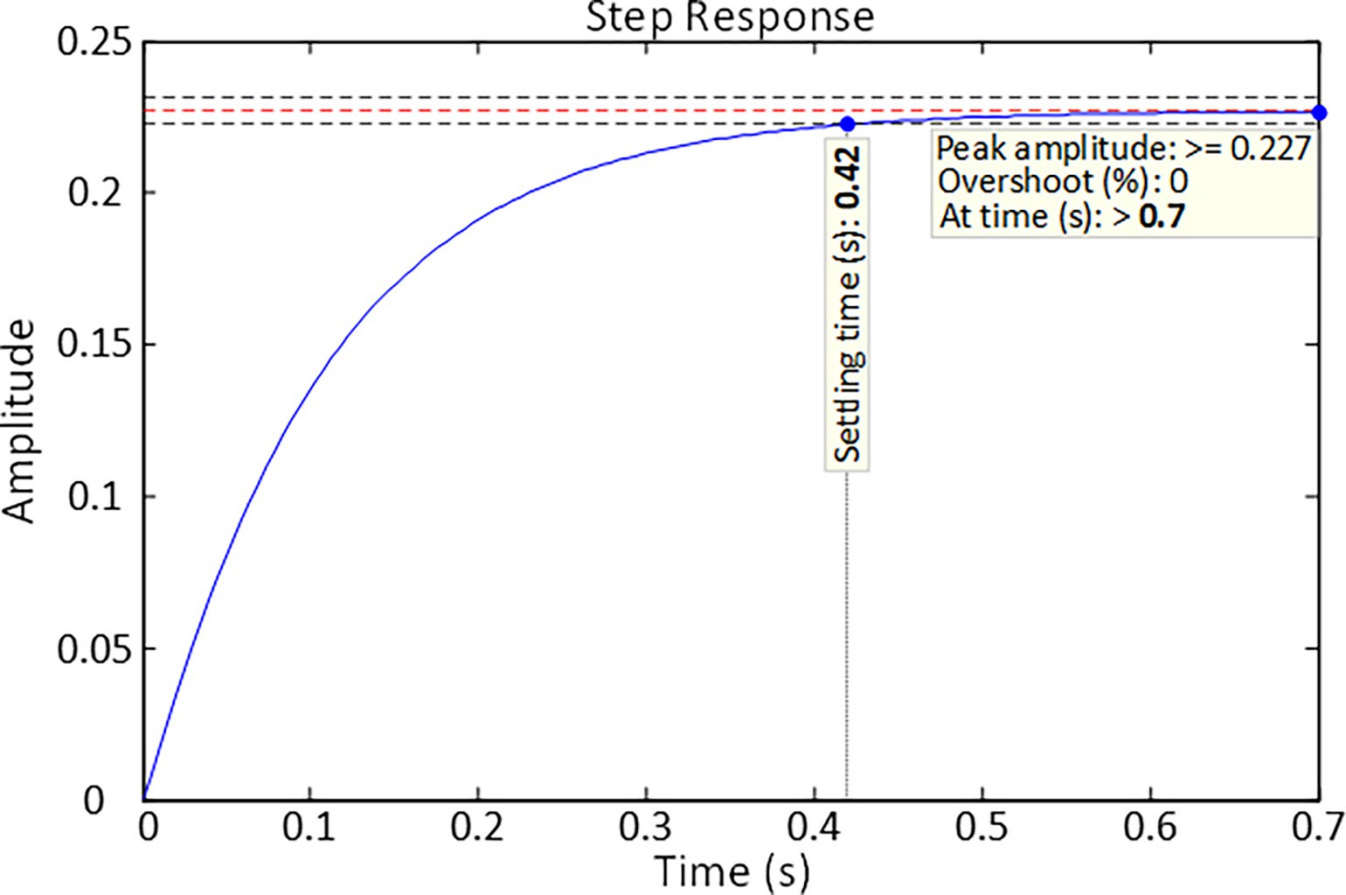
Step response of uncontrolled DFIG-based wind turbine system.

The step response of an uncontrolled DFIG-based wind turbine system based on the presented transfer function is displayed in Fig 5. As can be seen, the system has a settling time of 0.42 *s* with a 0% overshoot at time *t*>0.7 *s*. These figures must be further improved with the aid of an appropriate control scheme. Therefore, the following section explains the structure of the controller adopted for this study in order to increase the performance of the systems under consideration.

### Fundamentals of the adopted controller

This paper adopts a proportional-integral-derivative (PID) controller for the control of different high-order engineering systems. A PID controller has the following form where the gains known as proportional, integral, and derivative are denoted by *K*_*P*_, *K*_*I*_, and *K*_*D*_, respectively [47].


$$C(s)=K_{P}+\frac{K_{I}}{s}+K_{D}s$$ (14)

**Fig 6:**
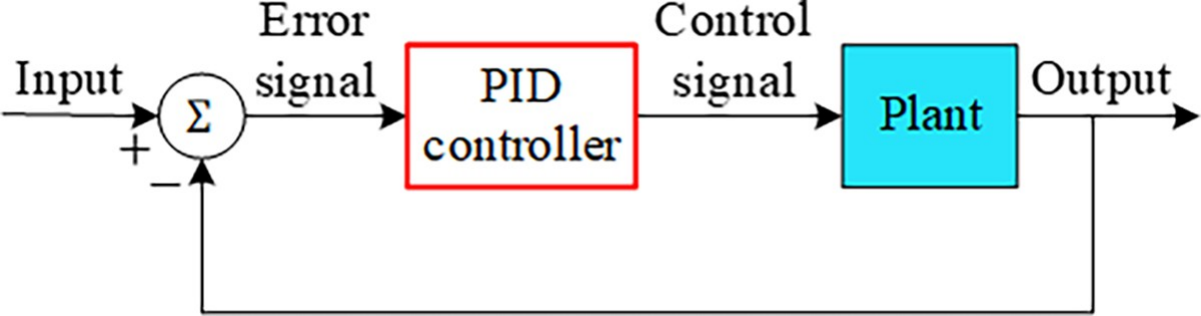
Block diagram of feedback control system with PID controller.

The block diagram illustrated in Fig 6 shows how a PID controller achieves the stated task for any plant in a feedback control system.

PID controller is important in control systems as it helps to regulate and stabilize a process by continuously adjusting the input to maintain a desired output. It is therefore feasible to adopt the PID controller as an effective mechanism to improve the performance of the high-order engineering problems discussed in the previous section. The next section discusses how this controller can be used in combination with the proposed h-ASPSO algorithm as a highly effective approach.

### Proposed novel design procedure

The design procedure for the implementation of the proposed algorithm to the investigated systems is explained in this section. Initially, the respective engineering problems must be described as minimization problems such that the optimization algorithm can be used to perform meaningful tasks. In terms of the description of the AVR system as a minimization problem, the following procedure is adopted in this study so that the parameters of the PID controller can be optimized. Firstly, the problem is represented as $\vec{X}=[x_{1},x_{2},x_{3}]=[K_{P},K_{I},K_{D}]$ and secondly the $ZLG(\vec{X})$ cost function [52], given in Eq (15), is adopted for appropriate optimization via the proposed hybrid h-ASPSO algorithm.


$$ZLG(\vec{X})=(1-e^{-\sigma })\times \left(\frac{OS}{100}+e_{ss}\right)+e^{-\sigma }\times (t_{s}-t_{r})$$ (15)

In here, *σ*, *OS*, *e*_*ss*_, *t*_*s*_ and *t*_*r*_ respectively denote a balancing coefficient, percent overshoot, error (steady state), settling and rise times. The $ZLG(\vec{X})$ cost function is a time-domain performance index that can be used to extract the optimal PID parameters since it includes the stability criteria of the system (overshoot, steady-state error, settling time, and rise time) [53]. This procedure is subjected to the constraints of overshoot less than 2% and settling time less than 0.5 *s* along with the following ranges of 0.01≤*K*_*P*_≤2, 0.01≤*K*_*I*_≤2 and 0.01≤*K*_*D*_≤2 for the PID controller parameters.

**Fig 7:**
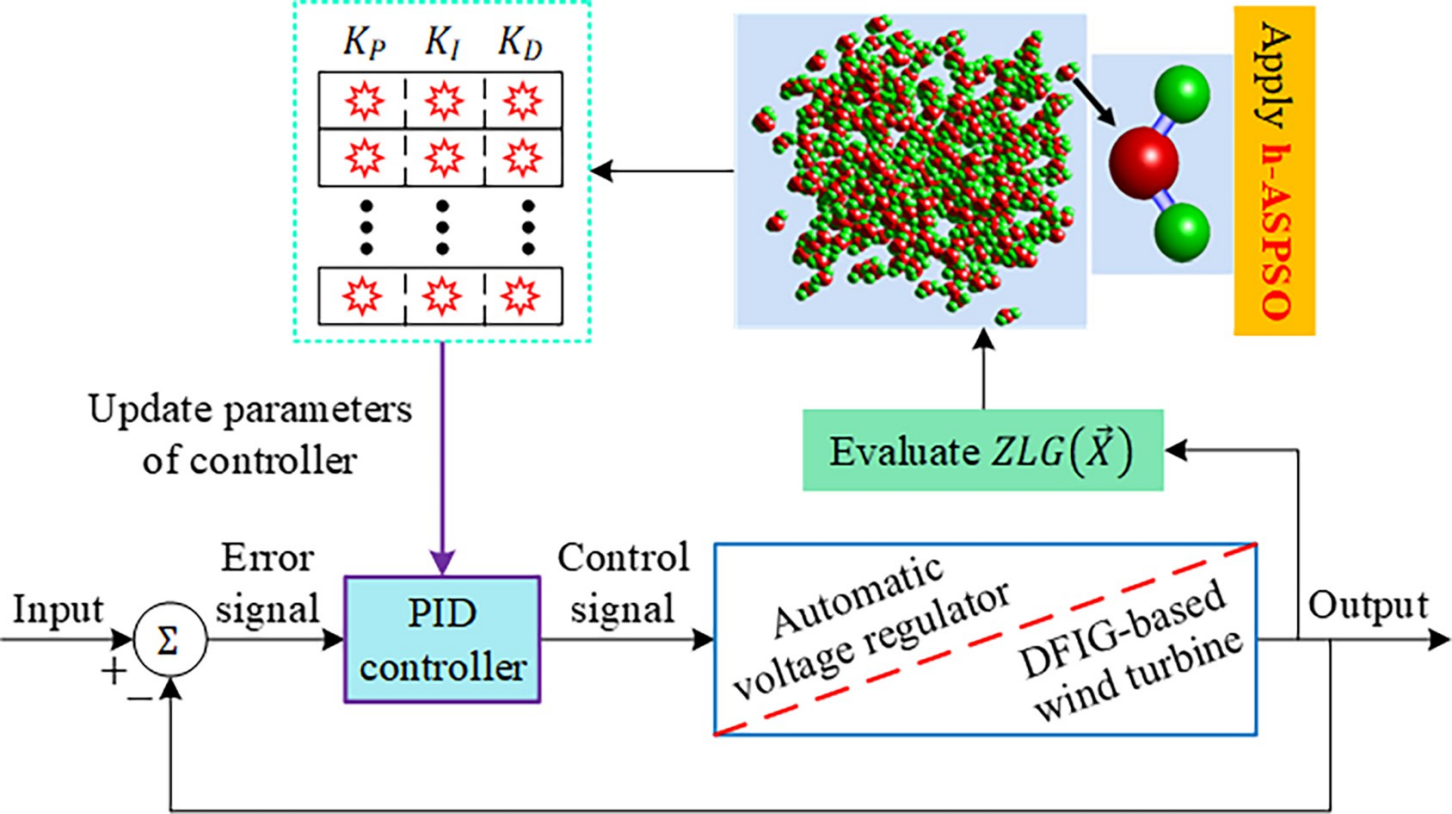
Implementation of the proposed design approach for optimizing AVR and DFIG-based wind turbine systems.

In case of the DFIG-based wind turbine system, the minimization problem is defined with the same manner, however, by considering the following constraints: overshoot less than 5% and settling time less than 0.25 *s* along with the following ranges of 0.1≤*K*_*P*_≤20, 1≤*K*_*I*_≤250 and 0.001≤*K*_*D*_≤1 for the PID controller parameters. Fig 7 demonstrates the implementation procedure of the proposed design approach for optimizing the AVR and DFIG-based wind turbine systems. This procedure is applied with the total iteration number of 50 and population size of 30. The respective algorithms are run for 30 runs in order to reach a fair conclusion for the performance. The comparative simulation results for the related systems are provided in the following section.

### Simulation results and discussions

This section provides comparative results regarding the transient response ability of the AVR and DFIG based wind turbine systems designed with the proposed h-ASPSO algorithm. The simulations were performed using the MATLAB/Simulink environment and were based on accurate mathematical models of the real-world systems that were under consideration. We also took great care to ensure that our simulation parameters were based on realistic values and that our results were consistent with existing literature on these systems. It is worth noting that the majority of related works reported in the literature have utilized simulation-based models to validate their proposed algorithms due to the complexity and time-intensive nature of performing experiments on actual systems. In this study, we have used a similar approach and validated our proposed algorithm using simulations of the actual systems in MATLAB/Simulink environment. We have conducted all the algorithms under the same number of fitness evaluations for fair comparison. We acknowledge that while simulation-based models have limitations, they still provide a valuable means of testing the performance of proposed algorithms in a controlled environment, which is essential in the development of efficient optimization techniques for complex engineering problems. Given these factors, the simulation results in this paper provide valuable insights into the performance of the proposed h-ASPSO algorithm for high-order engineering problems.

### Comparative results on automatic voltage regulator system

**Fig 8:**
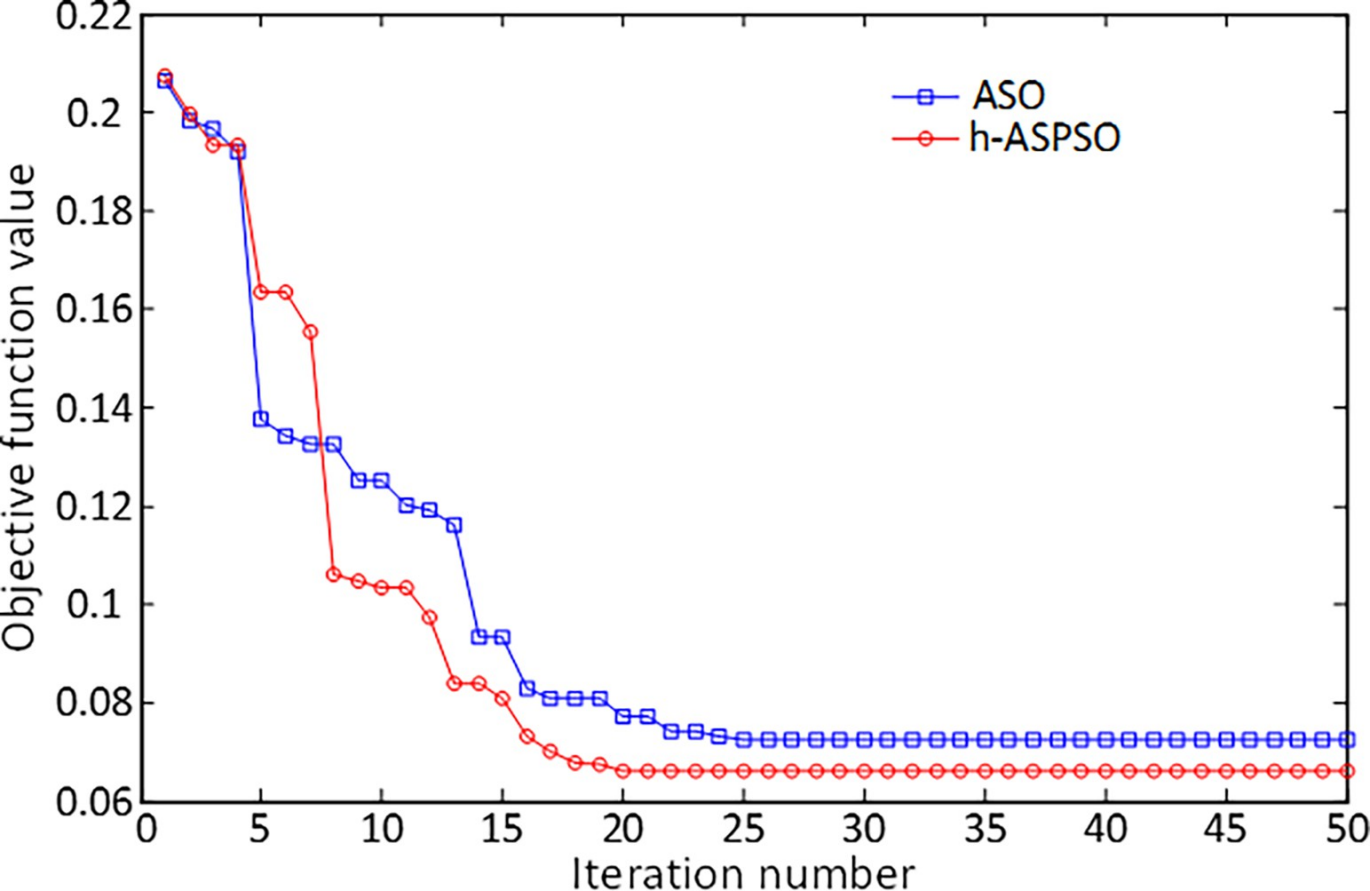
Convergence curves of ASO and h-ASPSO algorithms for AVR system.

The comparative convergence analysis illustrated in Fig 8 is presented in order to observe how well the proposed hybrid h-ASPSO based method finds the solution for the AVR system compared to the original version of ASO algorithm. h-ASPSO algorithm converges to the lowest objective function value, as depicted in the respective figure, in earlier iterations compared to the original ASO algorithm. This shows the more significant performance of h-ASPSO in terms of reaching the more convenient solution.

The performance of the h-ASPSO algorithm for AVR system is further demonstrated through statistical metrics of mean, standard deviation, minimum, maximum, and median values. Table 1 lists the values of those metrics. As can be observed, the proposed h-ASPSO algorithm is capable to reach lower values of the respective metrics signifying its better performance.

**Table 1:** Statistical metrics of $ZLG(\vec{X})$ objective function for AVR system.

Algorithm	Mean	Standard deviation	Minimum	Maximum	Median
h-ASPSO	6.7167E−02	6.9229E−04	6.6062E−02	6.8989E−02	6.7182E−02
ASO	7.3939E−02	1.1107E−03	7.2524E−02	7.6926E−02	7.3735E−02

In this study, genetic algorithm (GA) [54], symbiotic organisms search (SOS) algorithm [55], many optimizing liaisons (MOL) algorithm [56] are used to compare the performance of the ASO and h-ASPSO algorithms for the AVR system. Table 2 displays these algorithms and the gain parameters of the controller obtained via them. By substituting those values into Eq (14) and using the feedback controller scheme presented in Fig 6, the closed-loop transfer functions listed in Table 3 can be achieved for each related algorithm. The comparative assessments can be performed using those transfer functions.

**Table 2:** The obtained PID parameters for AVR system.

Algorithm	*K*_*P*_	*K*_*I*_	*K*_*D*_
h-ASPSO	0.6321	0.4158	0.2035
ASO	0.5855	0.4641	0.1897
GA [54]	0.6140	0.4560	0.1950
SOS [55]	0.5693	0.4097	0.1750
MOL [56]	0.5857	0.4189	0.1772

**Table 3:** Closed-loop transfer functions of optimization methods for AVR system.

Algorithm	Transfer function
h-ASPSO	$\frac{0.02035s^{3}+2.098s^{2}+6.363s+4.158}{0.0004s^{5}+0.0454s^{4}+0.555s^{3}+3.545s^{2}+7.321s+4.158}$
ASO	$\frac{0.01897s^{3}+1.956s^{2}+5.901s+4.641}{0.0004s^{5}+0.0454s^{4}+0.555s^{3}+3.407s^{2}+6.855s+4.641}$
GA [54]	$\frac{0.0195s^{3}+2.011s^{2}+6.186s+4.56}{0.0004s^{5}+0.0454s^{4}+0.555s^{3}+3.46s^{2}+7.14s+4.56}$
SOS [55]	$\frac{0.0175s^{3}+1.807s^{2}+5.734s+4.097}{0.0004s^{5}+0.0454s^{4}+0.555s^{3}+3.26s^{2}+6.693s+4.097}$
MOL [56]	$\frac{0.01772s^{3}+1.831s^{2}+5.899s+4.189}{0.0004s^{5}+0.0454s^{4}+0.555s^{3}+3.282s^{2}+6.857s+4.189}$

**Fig 9:**
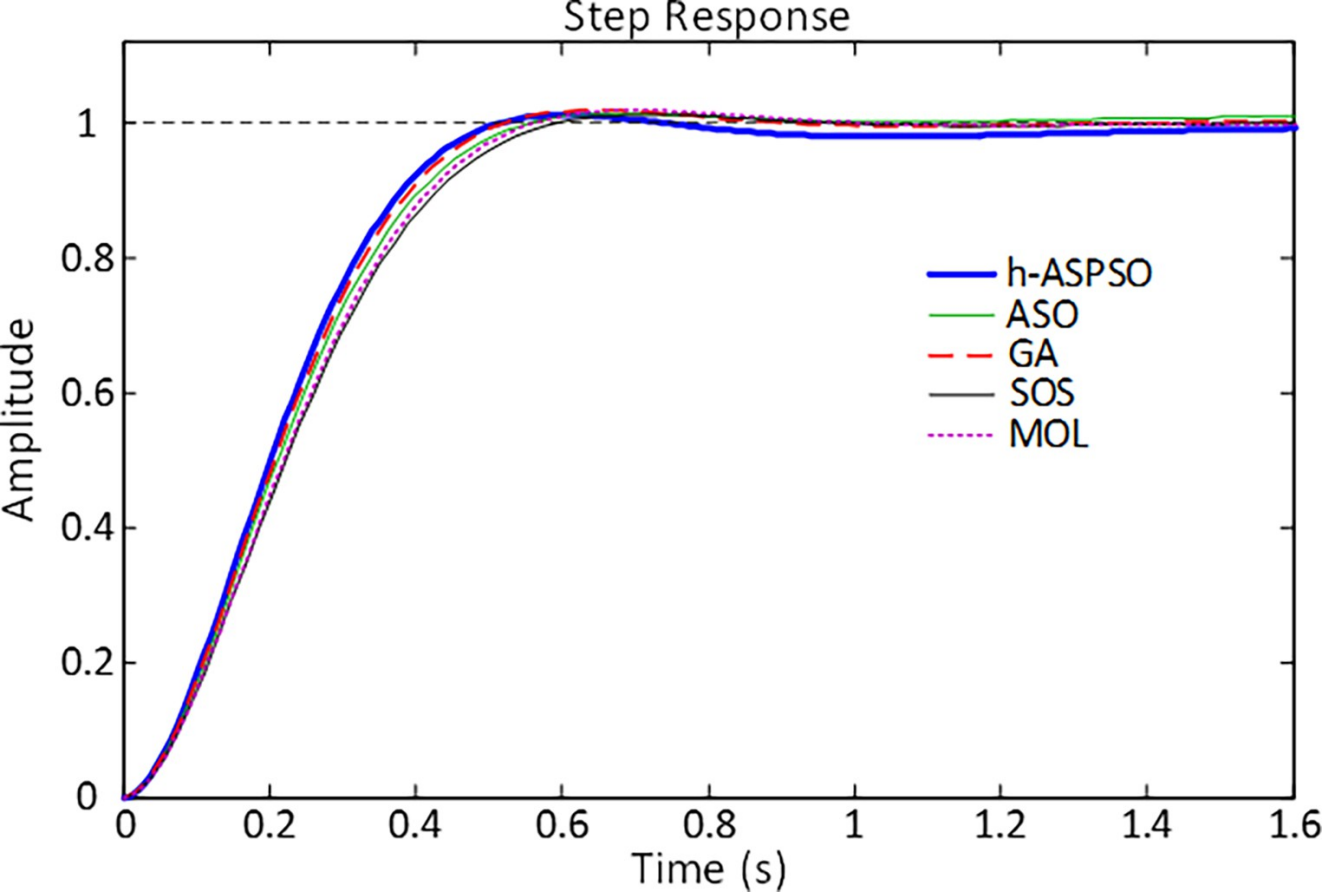
Step responses of PID controlled AVR system tuned by various algorithms.

Fig 9 illustrates the comparative step responses of different algorithms based PID controlled AVR systems. From the illustrative point of view the proposed h-ASPSO based PID design can be confirmed to have a more desirable behavior compared to the ASO, GA, SOS and MOL algorithms based PID design approaches. The desirable characteristics of the h-ASPSO based design approach can further be observed from the numerical values displayed in Table 4.

**Table 4:** Numerical values of time-domain performance indicators for AVR system.

Algorithm	Overshoot (%)	Rise time (s)	Settling time (s)	Peak time (s)
h-ASPSO	**1.2476**	**0.3097**	**0.4679**	**0.5993**
ASO	1.5023	0.3328	0.5042	0.6885
GA [54]	1.9793	0.3192	0.4784	0.6433
SOS [55]	1.2992	0.3529	0.5361	0.7231
MOL [56]	1.9547	0.3432	0.5154	0.7019

As shown with the bold values, the proposed approach has less overshoot, rise time (calculated as the time taken for the response to rise from 10% to 90% of its final value), settling time (calculated for a tolerance band of ±2%) and peak time, making h-ASPSO a more advanced technique for time-domain based performance of the AVR system.

**Fig 10:**
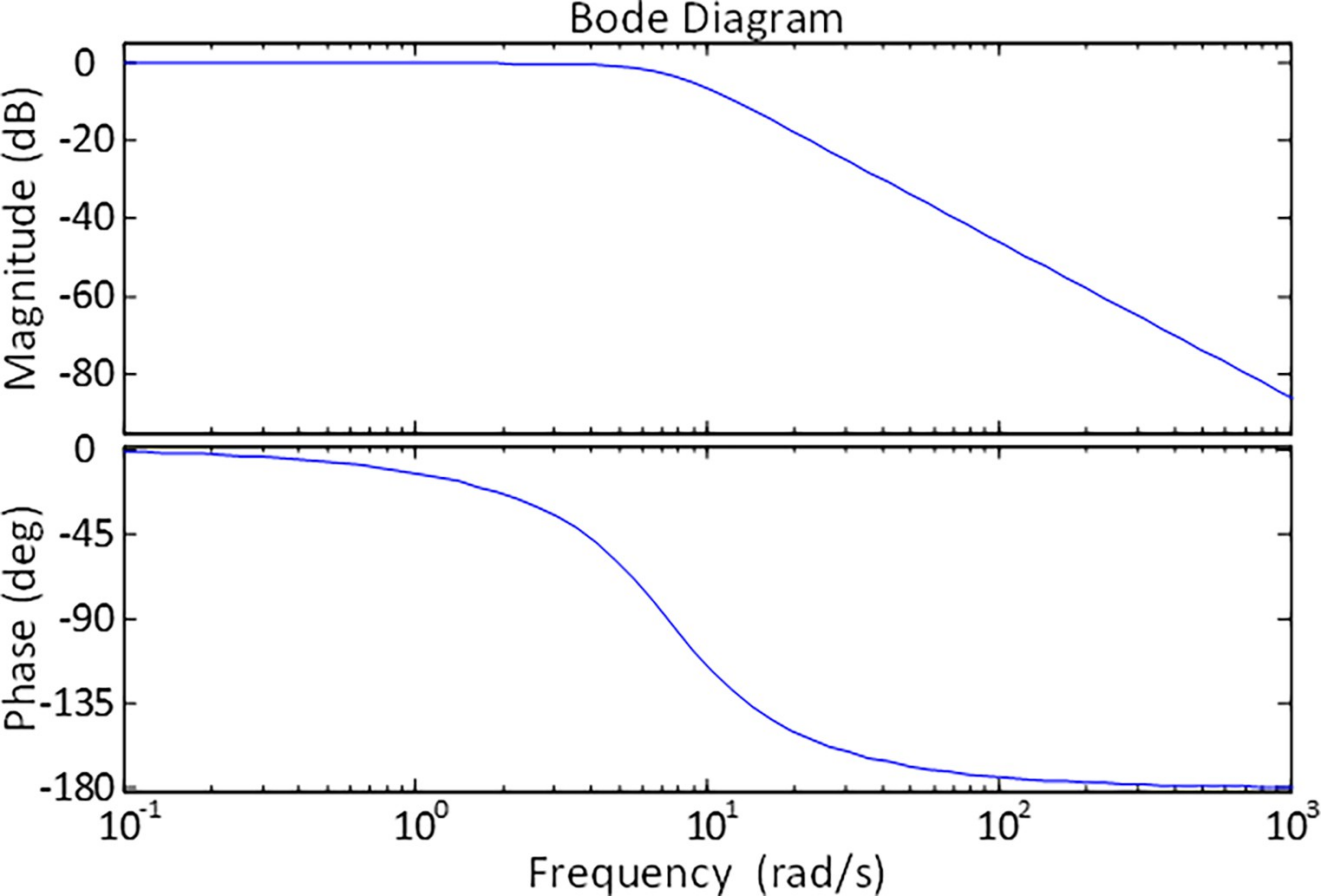
Bode plot of h-ASPSO optimized AVR system.

A stability check in the frequency domain is also conducted in this study to assess the stability of the AVR system in order to further investigate the stability-related results through another measure of stability criterion. In this regard, frequency response of the AVR system is also analyzed with the help of Bode diagram. Therefore, the Bode diagram of the h-ASPSO optimized AVR system is presented in Fig 10. Frequency-domain performance indicators of AVR system for h-ASPSO algorithm can be obtained as infinite for gain margin, 180° for phase margin and 7.1861 *rad*/*s* for bandwidth. As apparent from those results, the proposed h-ASPSO exhibits good results in terms of gain margin, phase margin and bandwidth.

### Comparative results on wind turbine system

**Fig 11:**
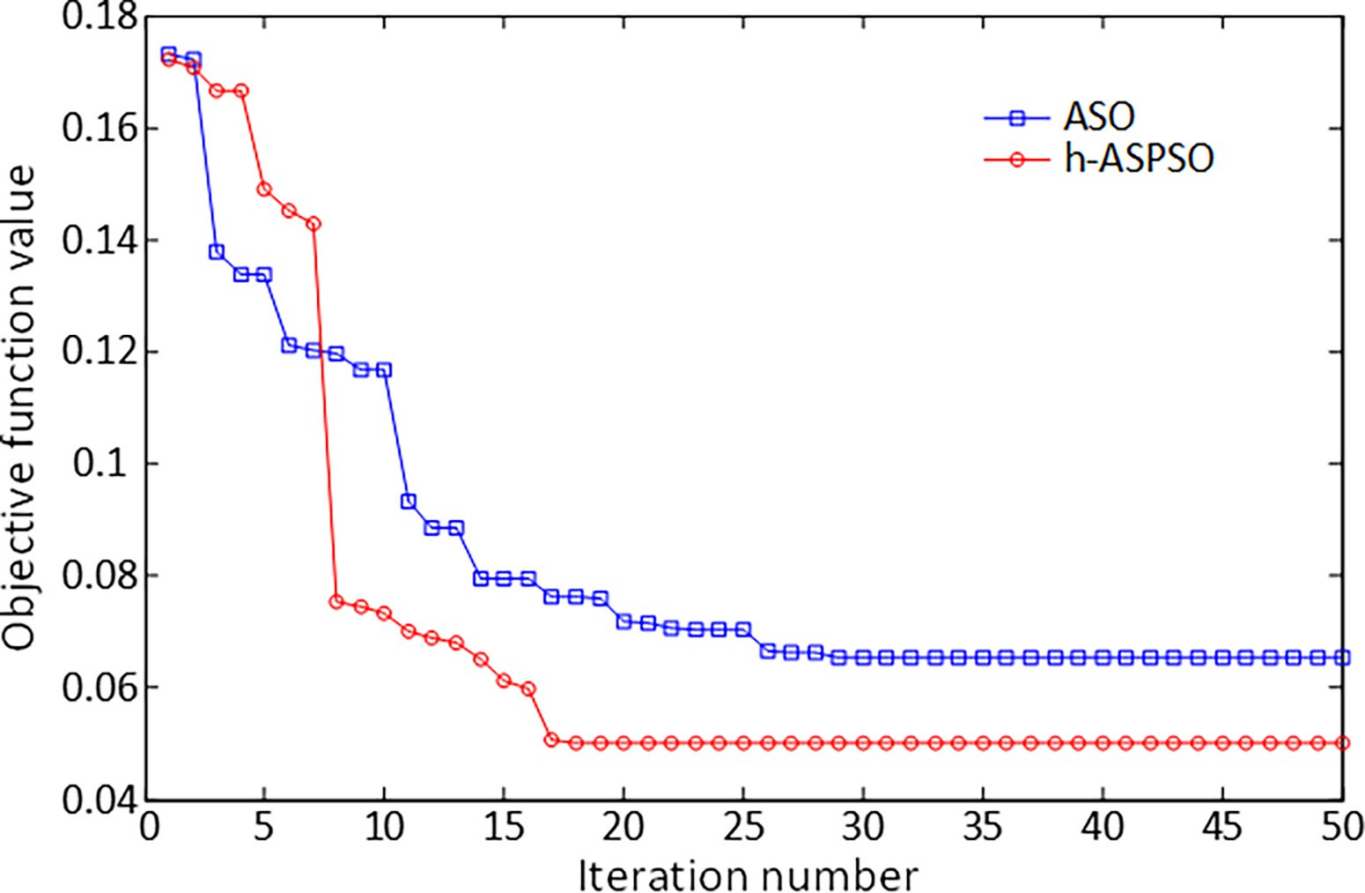
Convergence curves of ASO and h-ASPSO algorithms for DFIG-based wind turbine system.

The comparative convergence analysis illustrated in Fig 11 is presented in order to observe how well the proposed hybrid h-ASPSO based method finds the solution for DFIG-based wind turbine system compared to the original version of ASO algorithm. h-ASPSO algorithm converges to the lowest objective function value, as depicted in the respective figure, in earlier iterations compared to the original ASO algorithm. This shows the more significant performance of h-ASPSO in terms of reaching the more convenient solution.

The performance of the h-ASPSO algorithm for DFIG-based wind turbine system is further demonstrated through statistical metrics of mean, standard deviation, minimum, maximum, and median values. Table 5 lists the values of those metrics. As can be observed, the proposed h-ASPSO algorithm is capable to reach lower values of the respective metrics signifying its better performance.

**Table 5:** Statistical metrics of $ZLG(\vec{X})$ objective function for DFIG-based wind turbine system.

Algorithm	Mean	Standard deviation	Minimum	Maximum	Median
h-ASPSO	5.0921E−02	5.3738E−04	5.0075E−02	5.2293E−02	5.0879E−02
ASO	6.6901E−02	1.1249E−03	6.5371E−02	6.9339E−02	6.6551E−02

In this study, reptile search algorithm (RSA) [57], gravitational search algorithm (GSA) [58] and particle swarm optimization (PSO) [58] are used to compare the performance of the ASO and h-ASPSO algorithms for PID controlled DFIG-based wind turbine system. Table 6 displays these algorithms and the gain parameters of the controller obtained via them. By substituting those values into Eq (14) and using the feedback controller scheme presented in Fig 6, the closed-loop transfer functions listed in Table 7 can be achieved for each related algorithm. The comparative assessments can be performed using those transfer functions.

**Table 6:** The obtained PID parameters for DFIG-based wind turbine system.

Algorithm	*K*_*P*_	*K*_*I*_	*K*_*D*_
h-ASPSO	13.6145	124.9119	0.08982
ASO	10.0207	109.2884	0.1122
RSA [57]	18.7151	227.6068	0.08735
GSA [58]	16.9400	242.4000	0.1191
PSO [58]	6.2392	111.4924	0.0099

**Table 7:** Closed-loop transfer functions of optimization methods for DFIG-based wind turbine system.

Algorithm	Transfer function
h-ASPSO	$\frac{2.91e-05s^{8}-0.1528s^{7}-236.3s^{6}+6.771e05s^{5}+7.809e08s^{4}+5.522e11s^{3}+8.859e13s^{2}+3.593e15s+2.723e16}{2.91e-05s^{8}+0.8472s^{7}+2104s^{6}+9.347e06s^{5}+5.571e09s^{4}+3.252e12s^{3}+2.156e14s^{2}+4.553e15s+2.723e16}$
ASO	$\frac{3.635e-05s^{8}-0.1931s^{7}-283s^{6}+8.625e05s^{5}+9.204e08s^{4}+6.37e11s^{3}+7.538e13s^{2}+2.731e15s+2.382e16}{3.635e-05s^{8}+0.8069s^{7}+2057s^{6}+9.532e06s^{5}+5.71e09s^{4}+3.337e12s^{3}+2.024e14s^{2}+3.691e15s+2.382e16}$
RSA [57]	$\frac{2.83e-05s^{8}-0.1468s^{7}-239.3s^{6}+6.454e05s^{5}+8.024e08s^{4}+5.789e11s^{3}+1.143e14s^{2}+5.218e15s+4.962e16}{2.83e-05s^{8}+0.8532s^{7}+2101s^{6}+9.315e06s^{5}+5.592e09s^{4}+3.279e12s^{3}+2.413e14s^{2}+6.178e15s+4.962e16}$
GSA [58]	$\frac{3.859e-05s^{8}-0.2029s^{7}-311.4s^{6}+9.004e05s^{5}+1.027e09s^{4}+7.245e11s^{3}+1.125e14s^{2}+4.905e15s+5.284e16}{3.859e-05s^{8}+0.7971s^{7}+2029s^{6}+9.57e06s^{5}+5.817e09s^{4}+3.424e12s^{3}+2.395e14s^{2}+5.865e15s+5.284e16}$
PSO [58]	$\frac{3.208e-06s^{8}-0.0153s^{7}-34.31s^{6}+6.325e04s^{5}+1.233e08s^{4}+9.717e10s^{3}+3.419e13s^{2}+1.918e15s+2.431e16}{3.208e-06s^{8}+0.9847s^{7}+2306s^{6}+8.733e06s^{5}+4.913e09s^{4}+2.797e12s^{3}+1.612e14s^{2}+2.878e15s+2.431e16}$

**Fig 12:**
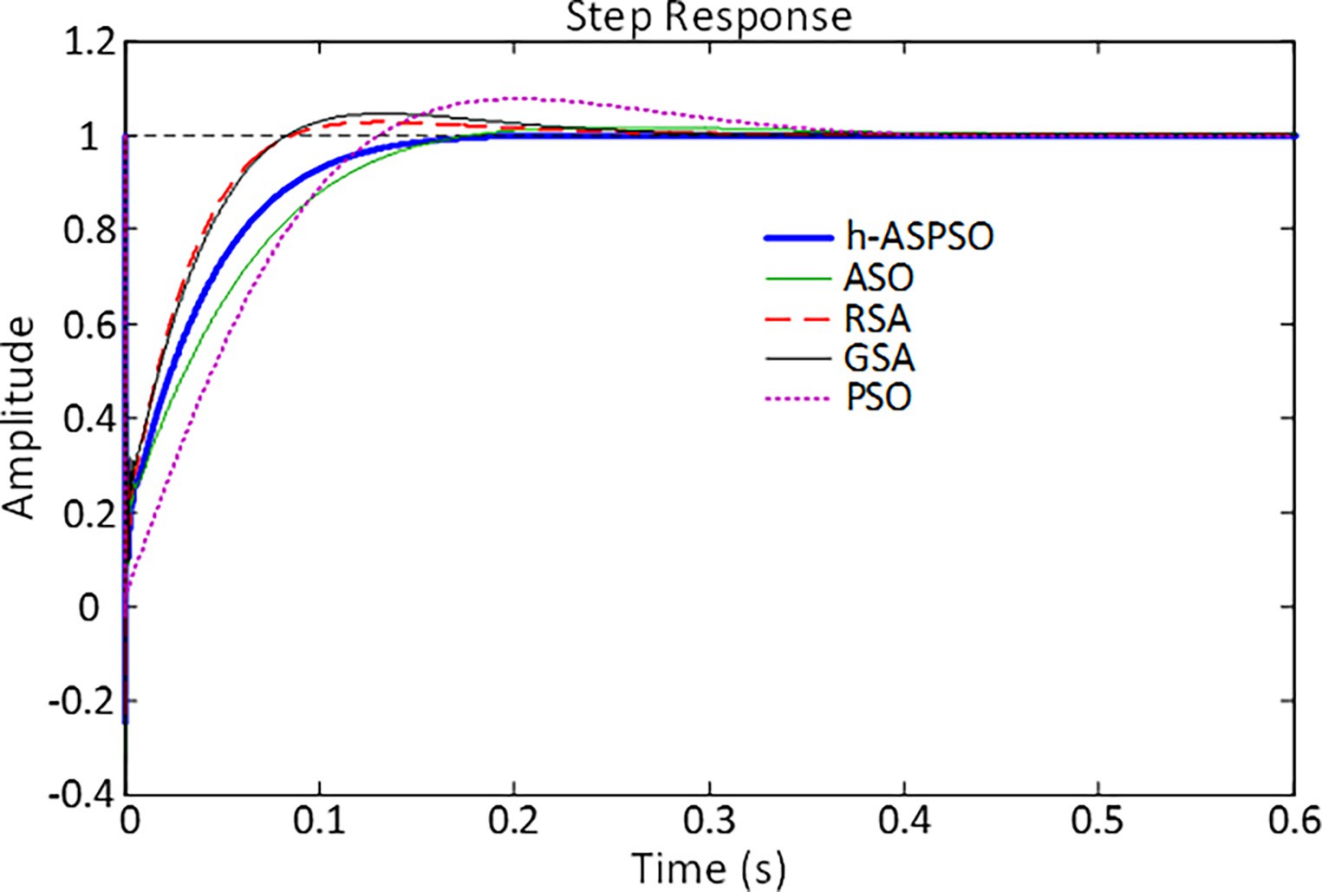
Step responses of PID controlled DFIG-based wind turbine system tuned by various algorithms.

Fig 12 illustrates the comparative step responses of different algorithms based PID controlled and DFIG-based wind turbine systems. From the illustrative point of view the proposed h-ASPSO based PID design can be confirmed to have a more desirable behavior, due to smoother characteristics, compared to the ASO, RSA, GSA and PSO algorithms based PID design approaches. The desirable characteristics of the h-ASPSO based design approach can further be observed from the numerical values displayed in Table 8. As shown with the bold values, the proposed approach has zero overshoot, rise time (calculated as the time taken for the response to rise from 10% to 90% of its final value) and peak time along with less settling time (calculated for a tolerance band of ±2%), making h-ASPSO a more advanced technique for time-domain based performance of the PID controlled and DFIG-based wind turbine system.

**Table 8:** Numerical values of time-domain performance indicators for DFIG-based wind turbine system.

Algorithm	Overshoot (%)	Rise time (s)	Settling time (s)	Peak time (s)
h-ASPSO	**0**	**0**	**0.1361**	**0**
ASO	1.7031	**0**	0.1484	0.2513
RSA [57]	2.7713	**0**	0.1570	0.1317
GSA [58]	4.6206	**0**	0.1970	0.1330
PSO [58]	7.6927	**0**	0.3338	0.2024

**Fig 13:**
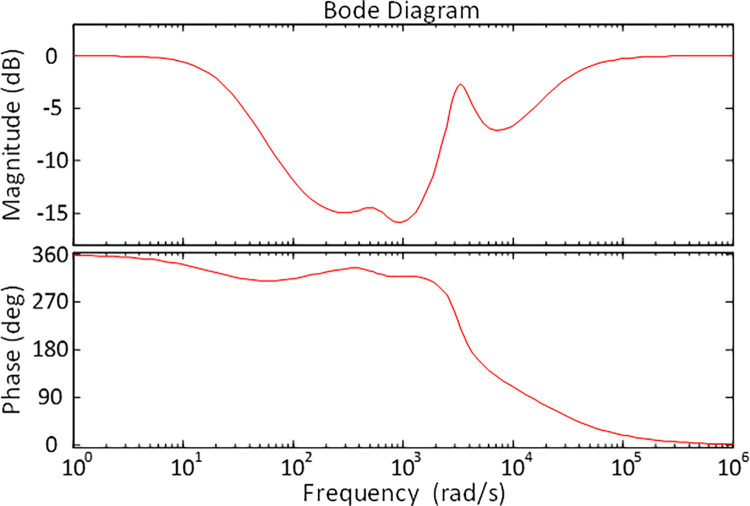
Bode plot of h-ASPSO optimized DFIG-based wind turbine system.

A stability check in the frequency domain is also conducted in this study to assess the stability of the DFIG-based wind turbine system in order to further investigate the stability-related results through another measure of stability criterion. In this regard, frequency response of the DFIG-based wind turbine system is also analyzed with the help of Bode diagram. Therefore, the Bode diagram of the h-ASPSO optimized DFIG-based wind turbine system is presented in Fig 13. Frequency-domain performance indicators of DFIG-based wind turbine system for h-ASPSO algorithm can be obtained as 4.3063 *dB* for gain margin, 180° for phase margin and 24.4168 *rad*/*s* for bandwidth. As apparent from those results, the proposed h-ASPSO exhibits good results in terms of gain margin, phase margin and bandwidth.

## Conclusion

Metaheuristic algorithms have gained popularity in recent years due to their effectiveness in solving complex optimization problems, especially in engineering design applications. However, no single algorithm can optimally solve all problems. The development of more efficient algorithms continues to be an important research area, with the potential to solve challenging real-world problems. The atom search optimization algorithm has shown great promise in solving real-world problems, but it also has some limitations that need to be addressed. To enhance its performance, researchers have proposed different approaches, including hybridizing it with other optimizers or incorporating stochastic operators. Achieving a balance between exploration and exploitation remains a challenge, and there is a need to establish an effective balance strategy for optimization problems. In this context, the proposed hybrid atom search particle swarm optimization algorithm combines the strengths of atom search optimization and particle swarm optimization to achieve a more effective balance between exploration and exploitation stages, leading to improved search efficiency. The efficacy of this algorithm has been demonstrated in improving the time-domain performance of two different high-order real-world engineering problems. In this context, proportional-integral-derivative controlled automatic voltage regulator and doubly fed induction generator-based wind turbine systems are used. The results confirmed the more promising efficacy of the proposed algorithm for the related high-order systems.

The comparative evaluation of optimization algorithms for high-order engineering problems demonstrates the potential of the hybrid atom search particle swarm optimization algorithm in improving the time-domain performance of high-order real-world engineering systems. In the proportional-integral-derivative controlled automatic voltage regulator system, hybrid atom search particle swarm optimization technique exhibits a lower percentage of overshoot (1.2476%), faster rise time (0.3097 *s*), settling time (0.4679 *s*), and peak time (0.5993 *s*) compared to other available techniques based on genetic algorithm, symbiotic organisms search algorithm, many optimizing liaisons algorithm and atom search optimization algorithm. Similarly, in the proportional-integral-derivative controlled and doubly fed induction generator-based wind turbine system, hybrid atom search particle swarm optimization technique shows significantly improved performance with zero overshoot, faster settling time (0.1361 *s*), and zero peak time compared to other available techniques of reptile search algorithm, gravitational search algorithm, particle swarm optimization and atom search optimization. In both cases, the hybrid atom search particle swarm optimization algorithm outperforms competitive algorithms in terms of time-domain response. These results demonstrate the potential of the hybrid atom search particle swarm optimization algorithm to address complex optimization problems in various fields with improved search efficiency and effectiveness.

In the future, further research can be conducted to explore the potential of h-ASPSO algorithm for solving a wider range of complex optimization problems in various fields such as healthcare, finance, and transportation. Some possible future scopes include (1) investigating the performance of the h-ASPSO algorithm on a wider range of real-world engineering problems to establish its generalizability, (2) exploring the potential of hybridizing the h-ASPSO algorithm with other metaheuristic algorithms to further enhance its search efficiency and effectiveness, (3) extending the application of the h-ASPSO algorithm to multi-objective optimization problems to address complex real-world optimization problems that involve multiple objectives, and (4) investigating the scalability of the h-ASPSO algorithm to handle larger optimization problems with high-dimensional search spaces. These are only few potential avenues that we can provide as a roadmap for future research and development in this area, which can help to advance the field of metaheuristic algorithms and their applications in engineering design and optimization.
