# Expression of Concern: Effective PID controller design using a novel hybrid algorithm for high order systems

**DOI:** 10.1371/journal.pone.0355529

**Published:** 2026-08-12

**Authors:** The PLOS One Editors

The *PLOS One* Editors issue this Expression of Concern for this article [1] because of concerns identified about the peer review process.

Readers are advised to interpret the article [1] with caution. In following up on this matter, PLOS did not evaluate the article’s integrity or scientific validity.

The Editors do not have concerns regarding the authors’ conduct during the peer review process.
